# Adherence to inhaled corticosteroids in patients with asthma prior to and during the COVID-19 pandemic

**DOI:** 10.1038/s41598-023-40213-6

**Published:** 2023-08-11

**Authors:** Isabel Rodríguez, Juan Carlos López-Caro, Silvia Gonzalez-Carranza, Maria Elena Cerrato, Maria Mar De Prado, Francisca Gomez-Molleda, Margarita Pinel, Maria Teresa Saiz, Carmen Fuentes, Esther Barreiro, Miguel Santibáñez

**Affiliations:** 1 Centro de Salud de los Corrales de Buelna, Servicio Cántabro de Salud, Los Corrales de Buelna, Spain; 2 Centro de Salud Cotolino, Servicio Cántabro de Salud, Castro Urdiales, Spain; 3 Centro de Salud Torrekúa, Osakidetza-Servicio Vasco de Salud, Eibar, Spain; 4 Centro de Salud La Corredoria, Servicio de Salud del Principado de Asturias, Oviedo, Spain; 5 Centro de Salud Basurto, Osakidetza-Servicio Vasco de Salud, Bilbao, Spain; 6 Centro de Salud Dobra, Servicio Cántabro de Salud, Torrelavega, Spain; 7 Centro de Salud La Habana-Cuba, Osakidetza-Servicio Vasco de Salud, Vitoria, Spain; 8 Centro de Salud Liebana, Servicio Cántabro de Salud, Potes, Spain; 9 Centro de Salud Cicero, Servicio Cántabro de Salud, Gama, Spain; 10 Pulmonology Department-Muscle Wasting and Cachexia in Chronic Respiratory Diseases and Lung Cancer, IMIM-Hospital del Mar, CEXS, Pompeu Fabra University, PRBB, Barcelona, Spain; 11 Centro de Investigación en Red de Enfermedades Respiratorias (CIBERES), Instituto de Salud Carlos III (ISCIII), Barcelona, Spain; 12 Global Health Research Group, Dpto Enfermería, Universidad de Cantabria-Instituto de Investigación Marqués de Valdecilla (IDIVAL), Avda. Valdecilla, s/n., 39008 Santander, Cantabria Spain

**Keywords:** Diseases, Health care

## Abstract

The electronic prescription refill rate (EPRR) of 183 consecutive patients was determined over a 19-month retrospective study period, divided into 7 months PRE (Sep-19 to Mar-20) and 12 months POST pandemic (Apr-20 to Mar-21), in order to compare adherence to inhaled corticosteroids (ICS) in patients with asthma prior to and during the COVID-19 pandemic. Before the pandemic (PRE), an average of 0.58 inhalers/month were refill from the pharmacy; [SD 0.33], very similar to the 0.59 inhalers/month; [SD 0.34] retrieved during the 12 subsequent months since the pandemic (POST) (p = 0.768). EPRR showed no differences (p = 0.784). When EPRR was dichotomous or ordinal categorised no differences were found either (p = 0.851 and 0.928), even when McNemar's test was used (p = 0.949), with prevalences of nonadherence (EPRR < 80%) of 57 and 58% respectively. Our results do not support increased adherence to inhaler treatment in terms of EPRR, comparing before and since COVID-19 pandemic. Compliance with prescription remains suboptimal.

## Introduction

Low adherence to inhaled therapy in patients with asthma is associated with increased morbidity and mortality, and with a greater use of the health-care services. More than 50% of asthma patients do not follow their treatment with the inhaler correctly^1–8^, which may compromise their health status and disease prognosis.

On 31 December 2019, the authorities of the People's Republic of China, reported to WHO some cases of pneumonia of unknown aetiology in the Chinese city of Wuhan, a city located in the Chinese province of Hubei. A week later, it was confirmed to be a new coronavirus now named SARS-CoV-2. This virus causes a variety of clinical manifestations under the term COVID-19, including respiratory illnesses ranging from the common cold to severe pneumonia with respiratory distress syndrome, septic shock and multi-organ failure. Its rapid spread and transmission among the population led to the ongoing COVID-19 pandemic, with a number of non-pharmaceutical interventions known as lockdowns encompassing stay-at-home orders, curfews, quarantines, and other societal restrictions that were implemented in numerous countries around the world.

In response to the increasing number of cases of COVID-19, the Spanish Government, at its extraordinary council on Saturday 14 March 2020, declared a state of alarm by imposing a severe national stay-at-home order that came into effect at 00:00 h on Sunday 15 March. On 28 April, the Spanish De-escalation Plan was announced, consisting of four phases in which the national lockdown was gradually reduced. Finally, the state of alarm ended on 21 June 2020 and the country moved to what was called the "new normality".

In relation to case fatality rate (CFR) and vulnerable groups, at the onset of the Pandemic in Europe, the data from Italy corroborated the population groups previously identified as vulnerable (at higher risk of severe disease and death). These groups were clearly people over 70 years of age; as well as people with comorbidities such as hypertension, diabetes, cardiovascular diseases, chronic respiratory diseases (including asthma), immunosuppression and cancer. At that point in time (at the time of the national Spanish severe lockdown on March 2020), it is likely that there will have been some degree of apprehension among asthma patients about the possibility of being at increased risk of severe COVID-19^9–12^.

After the advances in epidemiological knowledge on clinical course of COVID-19 disease and vulnerable groups, the role of chronic obstructive pulmonary disease (COPD) as a factor associated with a worse clinical course and higher mortality due to COVID-19 has been strengthened, while that of asthma is more controversial^13,14^. To date, the available evidence supports that patients with mild to moderate asthma are not at increased risk of severe COVID-19 disease, although patients who require oral corticosteroids for asthma control or those with severe asthma exacerbation (with hospitalisation) would be at increased risk^15^.

In addition to the fear of more severe COVID-19 disease, insecurity about the symptoms between uncontrolled asthma was foreseeable in asthma patients, which can be confused with those of COVID-19. In this regard, fear of SARS-CoV-2 infection could have constituted a reason for improved adherence during the COVID-19 pandemic in patients with asthma, as it was documented in a questionnaire survey at a university hospital in Japan^16^. On the other hand, the Covid-19 pandemic affected hospitals around the world, which postponed or reduced non-emergency care. In some cases it was even necessary to set up field hospitals at peaks of the Covid-19 pandemic. It is reasonable to think that in this context of hospital overburdening, the patients with asthma would want to avoid an asthma exacerbation requiring a visit to the emergency room or admission to hospital.

The self-perception as a high-risk vulnerable population to COVID-19, the coincidence of symptoms between asthma and COVID-19, and the fear of having an asthma exacerbation in a health care system overcrowded by the pandemic, are three supporting reasons to hypothesise an increase in adherence to inhaled therapy in asthma patients.

In the United States (U.S), a 14.5% relative increase in mean daily medication adherence was reported among patients with asthma and COPD during the COVID-19 pandemic, from March-11 (when WHO declared pandemic) to the last 7 days of March 2020. In contrast, daily adherence was more stable from the first days of January to the first days of March 2020^17^. In the United Kingdom (U.K) adherence to inhaled corticosteroids (ICS) was compared in patients with asthma in a longer-term perspective for the years 2019 and 2020 (from January 2019 to January 2021) by using a ratio of ICS issued/expected for each patient, reporting similar medians of adherence in a continuous analytical strategy (median ratios of 54.8%), but with a proportion of patients meeting “good adherence” (≥ 75%) increasing from 33.9 to 42.0%^18^. It would support our rationale, at least in the U.S and U.K.

The objective of the study was to compare the adherence to ICS in asthma patients with ICS maintenance treatment, before and during the COVID-19 pandemic in Spain.

## Methods

### Study design and patients

This was a retrospective cohort, multicenter study. The inclusion criteria were as follows: (1) age ≥ 18 years; (2) diagnosis of asthma according to Global Initiative for Asthma (GINA) criteria^19^ at least 12 months before the baseline recruitment visit. (3) No exacerbations in the 4 weeks prior to study entry, (4) Ability to answer the study questionnaires: Asthma Control Test (ACT) and Test of Adherence to Inhalers (TAI), (5) signed informed consent. The exclusion criteria were: (1) Previous diagnosis of COPD confirmed, (2) Inability to use an inhaler due to physical or psychological limitations, (3) Patients who do not have an electronic prescription (no possibility of pharmacy records).

The study was conducted in accordance with the Declaration of Helsinki and was approved by the Clinical Research Ethics Committees of each province. Written informed consent was obtained from all participants. All personal data were anonymized.

A panel of 21 primary care physicians from three different provinces in Northern Spain (Cantabria, Asturias and Basque Country) were selected to participate in the study. Each physician was expected to enroll 10 consecutive patients during the recruitment period. Finally, information from 196 patients was obtained: Cantabria (10 recruiting physicians, 91 patients), Basque Country (5 recruiting physicians, 50 patients) and Asturias (6 recruiting physicians, 55 patients).

For the main objective purpose, only asthma patients in maintenance treatment with inhalers (ICS ± LABA) (n = 183) were included for the PRE-POST comparisons. A sensitivity analysis was lastly established, restricted to those patients who, in addition to meeting the aforementioned additional inclusion criteria, remained unchanged from their baseline regimen over the last 19 months (since September 2019) (n = 164).

### Data collection and variables

The baseline maintenance inhaler treatment regimen (ICS ± LABA) was collected for each patient, as recorded in the clinical history. From this information, the number of inhaler devices prescribed per month by the physician for each patient was obtained. In addition the number of inhaler devices dispensed by the pharmacy in each month of follow up was also obtained. The 19-month retrospective study period was divided into 7 months PRE (September-19 to March-20) and 12 pandemic months POST (April-20 to March-21). Adherence was then assessed by the electronic prescription refill rate (EPRR) in every single patient (calculated by dividing the number of prescriptions dispensed at pharmacies by the total number of prescriptions in the study period and multiplying by 100%). In accordance with standard practice^3,7,20^, the refill threshold for good adherence was set at ≥ 80% and a patient with an EPRR < 80% was considered nonadherent. This nonadherence (EPRR < 80%) was classified by the following three ordinal categories: " High degree of nonadherence (EPRR 0–25%)", "Medium degree of nonadherence (EPRR 26–50)" and "Small degree of nonadherence (EPRR 50–79%)".

The following variables were also recorded: age, sex, severity level of the persistent asthma according to the Spanish Asthma Management Guideline (GEMA) (mild versus moderate)^21^, number of exacerbations in the last year, and type of device: Dry-Powder Inhaler (DPI) or metered-dose inhaler (MDI).

During the medical appointment, the patient's informed consent was obtained and all relevant demographic and clinical data were collected. All the participants completed the TAI (10 and 12 items) and the ACT.

### Statistical analysis

For categorical variables, proportions were estimated with their corresponding 95% confidence intervals (95% CI) and were comparison between independent groups were performed by using the Pearson's chi-square test, or Fisher's exact test as appropriate. For continuous variables, means with their standard deviation (SD) or medians and interquartile ranges (IQR) were estimated in the case of asymmetric distributions, and comparisons between independent groups were performed by using the Student’s T Test and Mann–Whitney’s U Test respectively.

The Student's t-test for related samples or Wilcoxon Signed Ranks Test when appropriate, were used to compare adherence in quantitative terms. McNemar's test was used to compare adherence in qualitative terms before (PRE) and since the COVID-19 pandemic (POST) (paired data).

A statistical significance level of 0.05 was considered for all hypothesis tests, and all tests were bilateral. Statistical analysis of the data was performed using SPSS 25.0 and Epidat 3.1 software.

### Ethics statement

The REFARMA study was first approved by the ethics committee of Cantabria with IR acting as principal investigator and subsequently by the ethics committees of the other provinces (CEIm-Cantabria 2020.470; CEIm-PA 2021.295; CEIm-E EOM2021035). The authors declare that they have complied with all relevant ethical standards. Written consent has been obtained from all patients. The REFARMA study does not involve animal testing.

## Results

### Description of the sample

Table 1 shows the clinical characteristics of the total patients recruited (n = 196). Four of the 196 patients (2%) did not have an ICS treatment prescribed and were therefore excluded from the final ICS adherence analysis. Nine of the 196 patients (4.6%) were excluded because they were prescribed only an as-needed ICS regimen as reliever therapy. Therefore, Table 1 also presents the clinical characteristics of the final sample of patients for the ICS adherence study (n = 183). The overall mean age was 49.77 years; [SD 17.58] in a range between 18 and 90 years. 57.9% were women (n = 106) and the rest men (n = 77, 42.1%).

**Table 1:** Baseline patient clinical characteristics.

	Total sample		In maintenance treatment with ICS	
	n = 196	%	n = 183	%
Age, mean [SD]	49.07	17.52	49.77	17.58
Gender				
Women	113	57.7	106	57.9
Men	83	42.3	77	42.1
Severity level of the persistent asthma according to GEMA				
Mild	104	53.1	91	49.7
Moderate	92	46.9	92	50.3
ACT, mean [SD]	20.09	4.08	20.14	4.10
ACT, median [IQR]	21	17–24	21	17–24
ACT ≥ 20 controlled asthma	119	60.7	112	61.2
ACT ≤ 19 uncontrolled asthma	77	39.3	71	38.8
Total control (25 points)	34	17.3	33	18
Good control (20–24 points)	85	43.4	79	43.2
Partial control (16–19 points)	50	25.5	47	25.7
Bad control (5–15 points)	27	13.8	24	13.1
Number of moderate-severe exacerbations without hospital admission (2020)				
0	147	75	138	75.4
1	25	12.8	23	12.6
2	13	6.6	12	6.6
3	7	3.6	6	3.3
4	1	0.5	1	0.5
6	1	0.5	1	0.5
7	2	1	2	1.1
Number of severe exacerbations with hospital admission (2020)				
0	192	98	179	97.8
1	4	2	4	2.2
Number of severe exacerbations with ICU admission (2020)				
0	195	99.5	182	99.5
1	1	0.5	1	0.5

*SD* standard deviation, *IQR* interquartile range, *GEMA* Spanish Asthma Management Guideline, *ACT* Asthma Control Test, *ICU* Intensive Care Unit.

Approximately half of the sample was diagnosed with mild persistent asthma (n = 91, 49.7%) and the rest with moderate persistent asthma (n = 92, 50.3%). Regarding the maintenance ICS treatment, 71.6% (n = 131) were on an ICS/LABA regimen (1/12 h), 27.9% (n = 51) on ICS/LABA (1/24 h), and the rest (3.3%) on ICS alone (1/12 h), with prescriptions of between 0.5 and 1.0 inhalers per month according to inhaler devices and prescribed dose. The most prevalent type of device was dry powder (84.7%).

The mean score in ACT was 20.14 points; [SD 4.10] with a median of 21, and an IQR between 17 and 24 points. Based on these scores, 44.8% of the total patients included had uncontrolled asthma (≤ 19 points), 49.8% had good control (20–24 points) and only 5.3% had total asthma control (25 points).

### Comparison of adherence before (PRE) and since the COVID-19 pandemic (POST)

In quantitative terms, during the 7 months of retrospective follow-up before the pandemic (September 2019 to March 2020) (PRE), an average of 0.58 inhaler devices/month were filled at the pharmacy; [SD 0.33], very similar to the 0.59 devices/month; [SD 0.34] retrieved during the 12 months of retrospective follow-up since the pandemic (April 2020 to March 2021) (POST) (p = 0.768). When comparing the inhalers retrieved to those prescribed, EPRR showed no differences with average compliances of 66.64% and 67.12% in the PRE and POST periods respectively (p = 0.784). See Table 2.

**Table 2:** Comparison of adherence in quantitative and qualitative terms before (PRE) and since the COVID-19 pandemic (POST).

	7 months of retrospective follow-up before the COVID-19 pandemic (Sep-19 to Mar-20) (PRE)		12-month retrospective follow-up since the COVID-19 pandemic (Apr-20 to Mar-21) (POST)		p value
	n = 183	%	n = 183	%	
No. of devices filled at the pharmacy/month, mean [SD]	0.58	0.33	0.59	0.34	0.768
No. of devices filled at the pharmacy/month, median [IQR]	0.57	0.29–0.86	0.5	0.33–0.92	0.926
EPRR, mean [SD]	66.64%	34.95%	67.12%	35.93%	0.784
EPRR, median [IQR]	57.14%	42.86–100%	66.67%	41.67–100%	0.864
Adherence (EPRR ≥ 80%)	79	43.2	77	42.1	0.851
Nonadherence (EPRR < 80%)	104	56.8	106	57.9	
High degree of nonadherence (EPRR 0–25%)	23	12.6	23	12.6	0.928
Medium degree of nonadherence (EPRR > 25 to 50%)	31	16.9	32	17.5	
Low degree of nonadherence (EPRR > 50 to < 80%)	50	27.3	51	27.9	

*SD* standard deviation, *IQR* interquartile range, *EPRR* electronic prescription refill rate.

In qualitative terms, during PRE period, EPRR was at least 80% in 43.2% of the sample, being the prevalence of nonadherence before the pandemic 56.83% (n = 104/183); 95% CI (49.38–64.28). In the subsequent pandemic period (POST), the prevalence of nonadherence was 57.92% (n = 106/183); 95% CI (50.50–65.35), p = 0.851. Ordinal categorisation of nonadherence showed no differences either (p = 0.928). See Table 2.

Table 3 presents a paired analysis of adherence in qualitative terms before and since the pandemic (PRE–POST). Nine of the 23 patients with High degree of nonadherence before the pandemic (EPRR 0–25%), moved to a better categories of “Medium or Low degree of nonadherence”. Eight of the 31 patients with Medium degree of nonadherence (EPRR > 25–50%) before the pandemic, moved to a better “Low degree of nonadherence” category, and 2 patients to an EPRR ≥ 80%. Eleven of the 50 patients with Low degree of nonadherence (EPRR > 50 to < 80%) before the pandemic, moved to an EPRR ≥ 80%. Overall, 30 patients (16.4%) qualitatively improved their adherence in the PRE-POST follow-up. On the other hand, there were patients whose adherence worsened: 7 patients changed from Medium to “High degree of nonadherence”. Eleven patients changed from low to “Medium or High degree of nonadherence” and finally 15 patients changed from being adherent (EPRR ≥ 80)% to nonadherent. Thus, in total 33 patients (18%) qualitatively worsened their adherence the PRE–POST follow-up. These differences were however not statistically significant in the McNemar's test (p = 0.949).

**Table 3:** Paired analysis of adherence in qualitative terms before (PRE) and since the COVID-19 pandemic (POST).

		Since the COVID-19 pandemic (Apr-20 to Mar-21) (POST)				Total
		≥ 80%	High	Medium	Low	
Before the COVID-19 pandemic (Sep-19 to Mar-20) (PRE)	Adherence (EPRR ≥ 80%)	64	0	1	14	79
	High degree of nonadherence (EPRR 0–25%)	0	14	8	1	23
	Medium degree of nonadherence (EPRR > 25 to 50%)	2	7	14	8	31
	Low degree of nonadherence (EPRR > 50 to < 80%)	11	2	9	28	50
Total		50	77	23	32	51

With regards to the sensitivity analysis, nineteen patients had a change in their medication regimen during the 19 months of retrospective follow-up. Table 4 presents the same results as Table 2 when restricting to patients with no change in their ICS regimen during the 19 months (n = 164). No statistically significant differences were found either, with p values even higher.

**Table 4:** Comparison of adherence before (PRE) and since the COVID-19 pandemic (POST) when restricting to patients with no change in their ICS regimen, during the 19 months of retrospective follow-up.

	7 months of retrospective follow-up before the COVID-19 pandemic (Sep-19 to Mar-20) (PRE)		12-month retrospective follow-up since the COVID-19 pandemic (Apr-20 to Mar-21) (POST)		p value
	n = 164	%	n = 164	%	
No. of devices filled at the pharmacy/month, mean [SD]	0.59	0.34	0.60	0.35	0.704
No. of devices filled at the pharmacy/month, median [IQR]	0.57	0.29–0.96	0.54	0.33–0.98	0.838
EPRR, mean [SD]	66.70%	34.25%	67.38%	35.15%	0.721
EPRR, median [IQR]	71.43%	42.86–100%	66.67%	41.67–100%	0.826
Adherence (EPRR ≥ 80%)	72	43.9	71	43.3	1
Nonadherence (EPRR < 80%)	92	56.1	93	56.7	
High degree of nonadherence (EPRR 0–25%)	20	12.2	22	13.4	0.938
Medium degree of nonadherence (EPRR > 25 to 50%)	27	16.5	25	15.2	
Low degree of nonadherence (EPRR > 50 to < 80%)	45	27.4	46	28.0	

*SD* standard deviation, *IQR* interquartile range, *EPRR* electronic prescription refill rate.

## Discussion

When comparing adherence to ICS based on the EFRR before and since the COVID-19 pandemic, our results in a primary care setting from the final sample of 183 asthma patients with a maintenance ICS regimen do not support greater adherence to treatment, neither in relation to the average number of devices filled at the pharmacy/month nor on the basis of % of adherence (EFRR) understood as the quotient between the average number of inhaler devices per month prescribed/filled. In qualitative terms, i.e. defining nonadherence based on the cut-off point < 80% and ordinal categorising the degree of nonadherence into High, Medium and Low, our results do not suggest greater adherence either.

Our sample comprises patients with mild to moderate persistent asthma on ICS ± LABA. When restricting to ICS in combination with LABA 1/12 h or 1/24 h, with at least one pack every two months and no change of their baseline regimen during the study period (19 months) in a sensitivity analysis, the results do not support either increased adherence.

Most publications report nonadherence prevalences > 50%, with no significant improvement in this aspect over the last few years^22,23^. In accordance, the prevalence of nonadherence based on an EFRR cut-off point < 80%, was 57% before the pandemic (PRE) and 58% in the subsequent 12 months pandemic period (POST). Compared to the latest Spanish published national studies, our prevalence is also in accordance with that reported by Entrenas et al.^24^. (60.90%), and lower than the 37.9% reported by Plaza et al.^7^, or the 25.0% and 39.6% reported by De Llano et al.^3^ in visit 2 for the One and Two Daily Doses regimens respectively, although in the latter two studies the period is restricted to 6 months before the COVID-19 pandemic.

Regarding limitations, as this was a retrospective study, we do not have TAI data prior to containment (Spanish stay-at-home order), so we could not compare PRE-POST adherence based on TAI results. In our sample, the mean in TAI (10 items) was 43.48; [SD 7.71] as measured in the recruitment visit, and the prevalence of non-adherence based on TAI results (≤ 49 points) was 73.2%. Regarding TAI 12 items results (and remembering that a single patient may have more than one type or pattern of nonadherence, and a patient with 50 points on the 10-item TAI may be an unconscious non-complier on the 12-item TAI): 69.9% of patients in our sample were patients who forgot to take their medication (sporadic non-compliance), i.e. with an unintentional passively inconsistent medication-taking behavior (forgetfulness or carelessness). 44.8% were patients who did not take their medication because they did not want to (Deliberate non-compliance), and 19.7% did not take their medication properly because they did not know the therapeutic regimen and how to use their inhaler device (Unconscious non-compliance). In none of these profiles did pharmacy refill increase, despite the fact that, as explained in the introduction section, in the beginning of the pandemic, chronic respiratory diseases such as asthma were associated with a higher CFR^10–13^, and even though in Spain, a severe national stay-at-home order was imposed at the onset of the Pandemic in Europe, with overburden of hospitals in some provinces such as Madrid.

Dispensing medication from the pharmacy does not necessarily mean that the patient uses it^7,23^. In this sense, the most reliable methods for measuring adherence would be direct electronic methods, not yet incorporated into daily clinical practice due to their price and/or complexity, based on the use of devices combined with the inhaler capable of recording the time of use or even the inspiratory flow^25,26^. Our study is based on monthly pharmacy dispensing of prescribed drugs, but does not allow for daily monitoring of their appropriate use. Anyway it is difficult to assume that the patient uses the medication if there is no record of the pharmacy dispensation (if the patient does not acquire the medication). Interesting data were reported by Kaye et al. for asthma and chronic obstructive pulmonary disease, by using these electronic medication monitors that even sent alerts to patients for missed doses^17^. They observed a 14.5% relative increase in mean daily controller for the last weeks of March 2020 after the first death in the US (Feb-25) and WHO declared pandemic (March-11). Up to our knowledge, no data is published on whether this increased adherence was continued throughout the rest of 2020 or 2021. Another study in the US compared retrospectively adherence in older adults with asthma, before (January to July 2019) and during the coronavirus disease (January to July 2020) without evaluating the period from August to December 2019. In this study adherence was measured using rates of proportion of days covered for dates instead of EFRR, and contrary to our hypothesis, adherence decreased when comparing 2019–2020 suggesting that medication adherence may have been negatively impacted by the COVID-19 pandemic^27^. Lastly, adherence to ICS was compared in the U.K from January 2019 to January 2021 by using a ICS medication possession ratio which can be considered similar to the EPRR since it was calculated as the number of doses of ICS issued/expected for each patient. The median levels of ICS adherence was 54.8% in 2019 as well as in 2020. Adherence was defined as good with a cut-off point ≥ 75% instead of 80%, and despite the identical median values, the proportion of patients who met the ≥ 75% threshold for “good adherence” increased from 33.9 to 42% in 2020 suggesting that the COVID-19 pandemic has witnessed an improvement to ICS^18^.

There are also studies that have used self-developed questionnaires instead of pharmacy data. In Japan, Fukutani et al. developed a one-time cross-sectional survey in 433 patients with asthma or COPD from January to March 2021 with some items related to adherence before and during the COVID-19 pandemic, reporting that adherence was significantly improved in both diseases during the COVID-19 pandemic, being the fear of infection the most common reason for this improved adherence^16^. In turkey, Yıldız et al. developed a questionnaire containing multiple-choice questions about adherence in asthma or COPD that was voluntarily applied to 82 physicians. Doctors thought that the main reason for nonadherence was the patients’ reluctance to be treated regularly and 43.2% of them perceived that adherence increased since the pandemic^28^.

Overall, improving adherence therefore remains a major problem in the asthma patients in particular and in the chronic respiratory patients in general, and it is therefore necessary to implement strategies in clinical practice to improve compliance. In this regard, the panelists of a recent Delphi study identified the modification of patients' beliefs, the training of professionals in adherence management and the personalisation of interventions as the most important strategies to improve adherence^29^. Interventions to improve adherence to ICS have also been the focus of a Cochrane review^30^ as well as different systematic reviews^31,32^. The type of prescription may also be related to adherence, with once-daily inhaled medications showing benefits in adherence when compared to twice-daily dosing^3^.

## Conclusions

Our results do not support increased adherence to ICS in terms of EPRR, comparing before and after the COVID-19 pandemic. Adherence remains suboptimal with a prevalence of nonadherent patients greater than 50%. The pandemic did not significantly influence adherence to ICS in patients with asthma.

### Supplementary Information

Supplementary Information.

## Data Availability

Data cannot be made publicly available in order to protect patients' privacy. The data are available on request from the University of Cantabria Archive (http://repositorio.unican.es/) for researchers who meet the criteria for access to confidential data. Requests may be sent to Professor Miguel Santibañez (miguel.santibanez@unican.es).
