# Supplementary material for: Adherence to inhaled corticosteroids in patients with asthma prior to and during the COVID-19 pandemic
Source: Sci Rep. 2023 Aug 11;13:13086. doi: 10.1038/s41598-023-40213-6 (PMC10421941; doi:10.1038/s41598-023-40213-6)
Supplement: Supplementary file 1 — Supplementary Information. [file 41598_2023_40213_MOESM1_ESM.docx]

**Supplementary Information**

ACINAR-REFARMA study group: Jose Miguel Alvarez Cabo; Sandra Arenal Barquin; Maria Concepción Astruga Tejerina; Helena Bermejo Ruiz; Maria Elena Cerrato Rodríguez; Maria Mar De Prado Taranilla; Francisco Borja del Rivero Sierra; Jose Francisco Egido Arroyo; Jose Ramón Fernández Fonfría; Carmen Fuentes Sainz; Francisca Gomez Molleda; Silvia Gonzalez Carranza; Ana Rosa Gutiérrez Rodríguez; Juan Carlos López Caro; Inmaculada Ortiz Lebaniegos; Ana María Piera Carbonel; Margarita Pinel Monge; Lorea Rivacoba Orbe; Isabel Rodriguez Marcos; Mariano Rodríguez Porres; Maria Teresa Saiz Careaga.
